# Demonstration of Fin-Tunnel Field-Effect Transistor with Elevated Drain

**DOI:** 10.3390/mi10010030

**Published:** 2019-01-07

**Authors:** Jang Hyun Kim, Hyun Woo Kim, Garam Kim, Sangwan Kim, Byung-Gook Park

**Affiliations:** 1 Inter-university Semiconductor Research Center, Department of Electrical and with the Department of Computer Engineering, Seoul National University, Seoul 151-744, Korea; neuburg@naver.com; 2 Department of Electrical and with the Department of Computer Engineering, Seoul National University, Seoul 151-744, Korea; hyunoo1218@naver.com (H.W.K.); kgr2487@gmail.com (G.K.); 3 Department of Electrical and Computer Engineering, Ajou University, Suwon 16944, Korea

**Keywords:** band-to-band tunneling, tunnel field-effect transistor, low operating power device, tunneling resistance, sub- threshold swing, ambipolar current, elevated drain

## Abstract

In this paper, a novel tunnel field-effect transistor (TFET) has been demonstrated. The proposed TFET features a SiGe channel, a fin structure and an elevated drain to improve its electrical performance. As a result, it shows high-level ON-state current (*I*_ON_) and low-level OFF-state current (*I*_OFF_); ambipolar current (*I*_AMB_). In detail, its *I*_ON_ is enhanced by 24 times more than that of Si control group and by 6 times more than of SiGe control group. The *I*_AMB_ can be reduced by up to 900 times compared with the SiGe control group. In addition, technology computer-aided design (TCAD) simulation is performed to optimize electrical performance. Then, the benchmarking of ON/OFF current is also discussed with other research group’s results.

## 1. Introduction

Numerous studies about tunnel field-effect transistor (TFET) have been performed by several research groups as a promising device for an ultra-low power operation [1,2,3,4]. In case of metal-oxide-semiconductor FETs (MOSFETs), there exist a theoretical limit of 60 mV/dec subthreshold swing (SS) at 300 K-temperature because their carrier injection is based on the thermionic emission [5,6]. On the other hand, TFETs are relatively independent to the Boltzmann distribution since the function tail is removed by forbidden gap and the band-to-band tunneling (BTBT) dominates the carrier injection from source to channel [7,8]. Thus, the SS can be reduced to less than 60 mV/dec at RT, which allows the supply voltage (*V*_DD_) to be decreased drastically, maintaining high ON-state current (*I*_ON_). In addition, its fabrication process is highly compatible with that of MOSFETs. In spite of these advantages, however, the TFETs have some technical issues to be employed for a real application. First, it suffers from low-level ON-state current which is mainly attributed to the high tunnel resistance at source-to-channel junction [9,10,11]. In order to solve this, the Ge material has been adopted for its low bandgap and direct BTBT tunneling [12]. However, It is difficult to make a heterojunction using Ge material [12]. Second, a BTBT at channel-to-drain junction increases OFF-state leakage current (*I*_OFF_); ambipolar current (*I*_AMB_). Since these issues degrade TFET circuit’s electrical performance such as operation speed and power consumption, they should be addressed [13,14,15,16].

**Figure 1:**
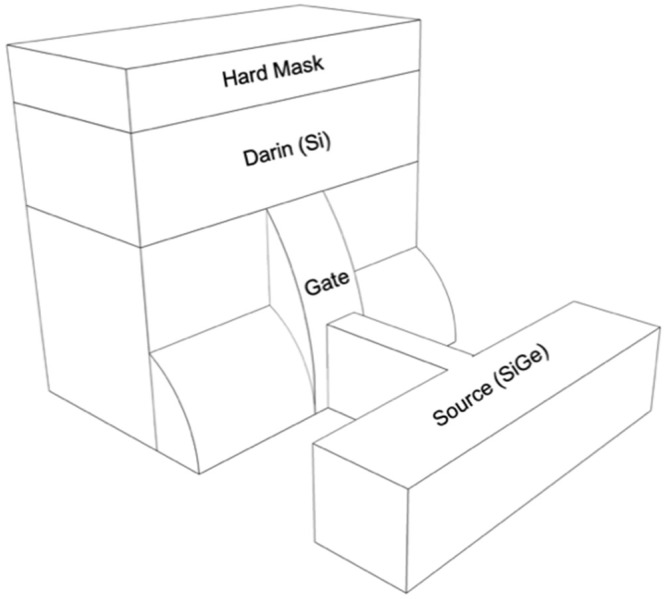
Structure of the proposed TFET. It is featured that SiGe fin structure with elevated drain region.

The purpose of this paper is to demonstrate a novel TFET which achieves larger *I*_ON_ and smaller *I*_AMB_ than that of conventional Si TFETs. As shown in Figure 1, the proposed TFET features a fin channel structure for improved gate controllability and a SiGe channel for higher *I*_ON_ as reducing tunnel resistance. In addition, in the proposed TFET, *I*_AMB_ can be suppressed with the help of relatively large Si band gap at drain. In addition, its feasibility for better performance is examined by technology computer-aided design (TCAD) simulation. Last of all, based on the measurement and optimized results, the benchmarking of ON/OFF current ratio (*I*_ON_/*I*_OFF_) and SS with the state-of-the-art TFETs is also discussed.

## 2. Device Fabrication

**Figure 2:**
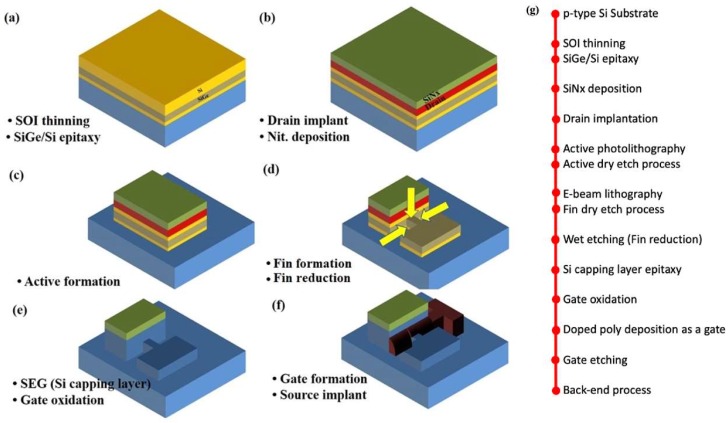
Fabrication process flow of the proposed TFETs. The flow graph summarize all the fabrication process briefly.

**Figure 3:**
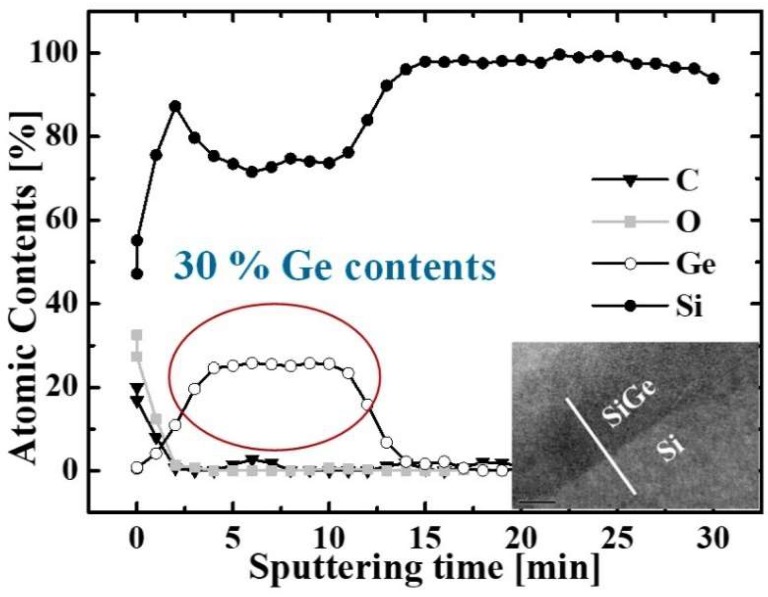
Auger electron spectroscopy (AES). The SiGe layer contains 30% Ge.

**Figure 4:**
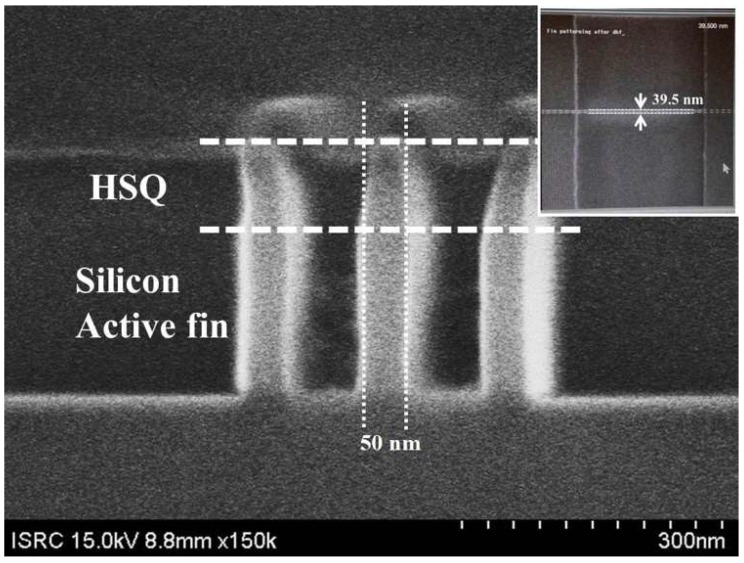
Cross-sectional view of scanning electron microscope (SEM) image which demonstrates Si fin etching. The inset shows top view of SEM image after fin etching and SC-1 reduction processes.

The key process steps for the proposed TFETs are described in Figure 2a First, silicon-on-insulator (SOI) thickness is decreased by using wet oxidation followed by SiO_2_ wet etching. Then, SiGe and Si layers are grown on the SOI substrate by metal organic chemical vapor deposition (MOCVD). The process condition is as follow: a gas mixture of H_2_ at 20 sccm, SiH_4_ at 20 sccm, and GeH_4_ at 90 sccm is used at 670 °C during 61 s for 300 Å-thick SiGe. Auger electron spectroscope (AES) and transmission electron microscope (TEM) image confirm a single crystalline Si_0.7_Ge_0.3_ is well grown on Si substrate (Figure 3) As ion implantation is performed at 10 keV-acceleration energy, 7°-tilted angle and 8 × 10^14^ ions/cm^2^-dose. Then, SiN_x_ is deposited by plasma-enhanced CVD (PECVD) as an etching mask during an active patterning (c). PECVD nitride is adopted since it is low temperature process with 400 °C and 20 s, in which the implanted dopants in the drain region can rarely diffuse. (d) Some part of Si on active regions are removed by photolithography and reactive ion etching (RIE) processes forming SiGe source and channel while the remaining Si on mesa becomes raised drain region. In case of channel, an additional patterning is conducted by mix-and-match process of e-beam lithography and photolithography to form 50 nm-width active fin (Figure 2d and Figure 4).

**Figure 5:**
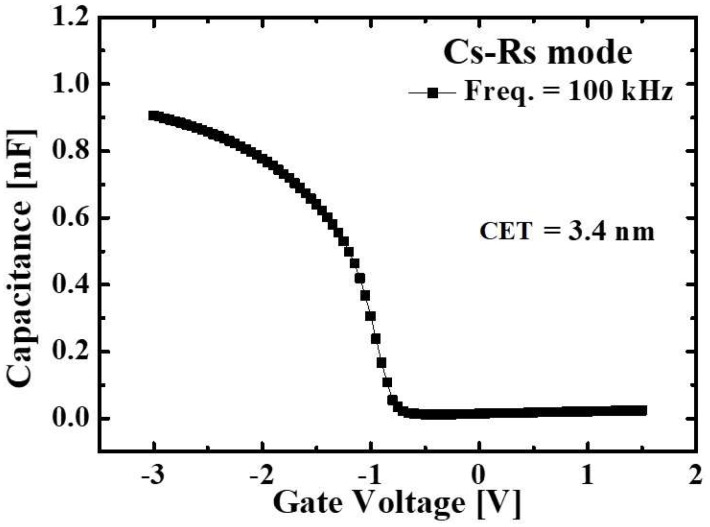
Measured C-V curves of MOS capacitors. The measured CET is 3.4 nm.

**Figure 6:**
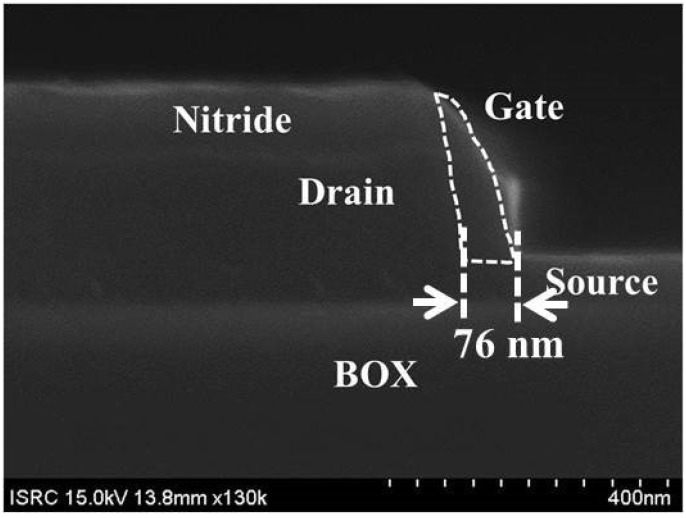
Cross-sectional SEM image which demonstrates raised drain and sidewall gate structure. The 76 nm-length gate self-aligning to the drain is well defined.

The SiGe/Si fin width is further reduced by standard cleaning-1 (SC-1) solution which consists of ammonium hydroxide (NH_4_OH), hydrogen peroxide (H_2_O_2_), and de-ionized wafer (H_2_O) [17,18]. The NH_4_OH:H_2_O_2_:H_2_O ratio is 1:8:64 in which the etching rate of the SiGe is ~0.85 nm/min. After 13 min process in the SC-1 solution, the SiGe fin width is reduced to 39.5 nm as shown in the inset of Figure 4. As shown in Figure 2e, an 1 nm-thick Si capping layer is deposited by selective epitaxy growth (SEG) followed by dry oxidation for a gate dielectric. It has been demonstrated that this process can efficiently prevent defects which could be induced between SiO_2_ and SiGe [19]. The capacitance equivalent thickness (CET) of gate dielectric is confirmed as 3.4 nm from the capacitance-voltage (C-V) curve shown in Figure 5. (f) For a short-channel gate, sidewall spacer technique is applied: n-type doped polycrystalline-Si (poly-Si) is deposited by low pressure CVD (LPCVD) and etched by Si RIE process after photolithography for a gate pad. As a result, ~76 nm-length gate is defined self-aligning to the drain (Figure 6). After that, BF_2_ implantation with 10 keV-acceleration energy, 7°-tilted angle and 8 × 10^14^ ions/cm^2^-dose is performed for a source region. The dopant activation is performed by rapid thermal process (RTP) with 900 °C and 5 s. Note that all processes for gate, source and drain formation are self-aligned to each other and can be compatible with state-of-the-art ultra-short channel technology. Finally, as a back-end-of line (BEOL), high plasma density (HDP) oxide is deposited as an interlayer dielectric (ILD) and metal layers (Ti/TiN/Al/TiN stacks) are deposited by physical vapor deposition (PVD) after contact formation. (g) Then, all of processes are summarized in the flow graph.

For the control samples, the planar Si and SiGe TFETs were fabricated. In the case of SiGe TFET, Si_0.7_Ge_0.3_ layers with a thickness of 30 nm are grown on SOI (100) substrates. The SOI layer is lightly p-doped (1 × 10^15^ cm^−3^) with a thickness of 70 nm. For additional comparison, the Si TFET is fabricated on a 100 nm-thick SOI wafer. The gate stack consists of 200 nm poly-Si layer and 3 nm SiO_2._ After gate patterning, source and drain region are defined through photolithography and ion implantation processes. The ion implantation and BEOL processes are same with the processes in proposed TFET.

## 3. Measurement and Results

**Figure 7:**
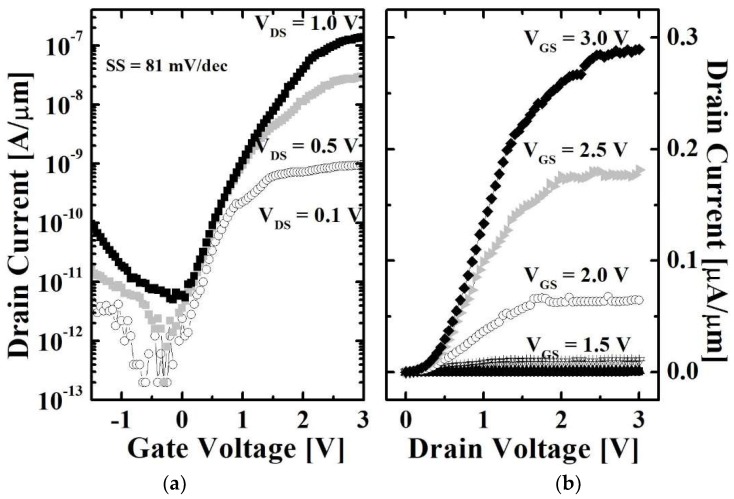
(**a**) Transfer and (**b**) output characteristics of proposed TFET.

Figure 7a shows the transfer characteristics of the proposed device with the various drain voltages (*V*_DS_s). The SS is extracted at *V*_DS_ of 0.1 V and a turn-ON voltage (*V*_turn-ON_) is defined as gate voltages (*V*_GS_) where BTBT first occurs. The *I*_OFF_ and *I*_ON_ are extracted when *V*_GS_ is *V*_turn-ON_ and gate overdrive (*V*_OV_ = *V*_GS_ − *V*_turn-ON_) is equal to 2 V, respectively. The minimum SS is 81 mV/dec and *I*_ON_/*I*_OFF_ is 2.8 × 10^4^. Figure 7b shows the output characteristics of the proposed TFET with the various *V*_GS_s. Note that, the conventional planar devices suffer from short channel effect (SCE) due to their weak gate controllability over the channel [20,21]. Generally, the SCE can be confirmed with drain induced current enhancement (DICE) in transfe curves and increase of saturation current in output characteristics. According to the measured results, however, there is no obvious SCE in the proposed TFET as shown in Figure 7a,b.

**Figure 8:**
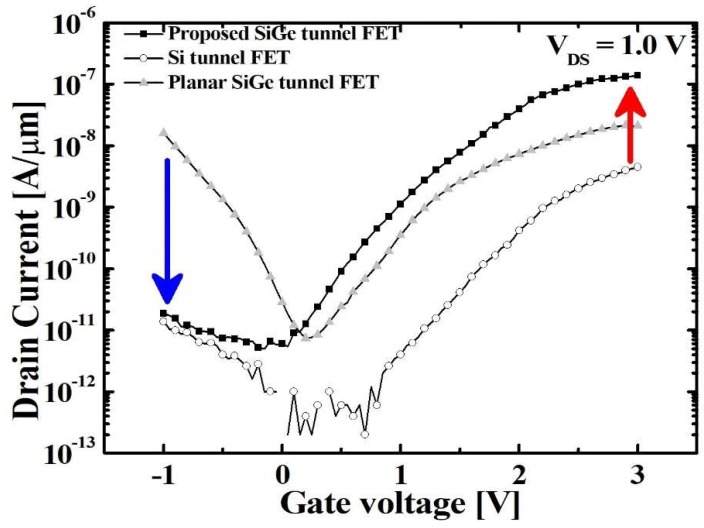
Transfer characteristics of the proposed TFET and conventional planar TFETs with Si and SiGe channels.

The proposed TFET’s electrical characteristics are compared with that for planar Si and SiGe TFETs as control groups. Figure 8 shows the transfer characteristics of both groups at 1.0 V-*V*_DS_. The SS and *I*_ON_, *I*_AMB_ and *I*_ON_/*I*_OFF_ are extracted from the curves and summarized in Table 1. The proposed TFET shows superior performance than the control ones in the several aspects. First, the SS of proposed device, which is measured at *V*_turn-ON_ is 81 mV/dec whereas 151 mV/dec and 87 mV/dec are measured in planar Si and SiGe TFETs, respectively. Second, the proposed TFET shows 139 nA/μm-*I*_ON_ which is 34 times and 5 times bigger than that for Si and SiGe TFETs, respectively. Last of all, the *I*_AMB_ can be reduced by up to 10^3^ times compared with the SiGe TFET. These results are attributed in part to the SiGe’s narrow bandgap at the source area and in part to the strong gate-to-channel coupling with the help of fin-structured channel [22]. In addition, the elevated drain area reduces the BTBT between the channel and the drain by Si bandgap [23].

**Figure 9:**
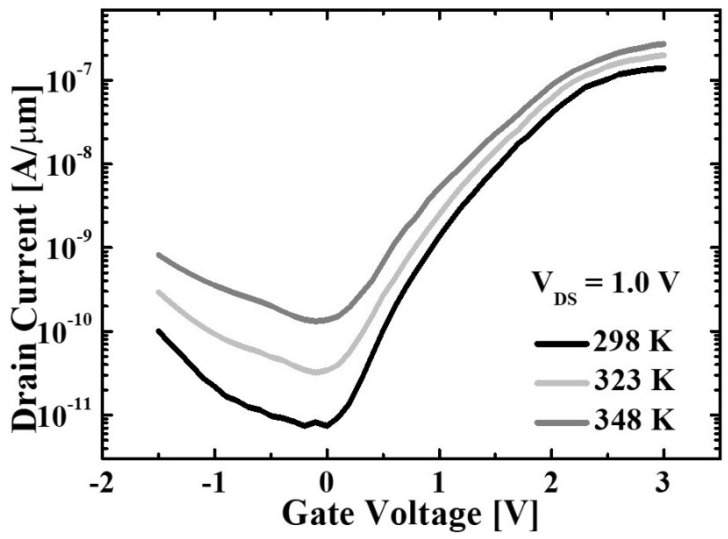
Transfer characteristics of the proposed TFET in the range of 298–348 K.

The proposed TFET has remarkable electrical characteristics as shown above. However, the *I*_OFF_ of proposed TFET near zero *V*_GS_ is higher than that of planar Si TFET (Figure 8). In order to confirm the mechanism precisely, transfer characteristics with various temperature are investigated. As shown in Figure 9, drain current (*I*_D_) is relatively independent to the *V*_GS_ at around 0 V while it increases rapidly as a function of temperature. The result confirms that this current is dominated by Shockley–Read–Hall (SRH) generation–recombination [24].

## 4. Discussion

**Figure 10:**
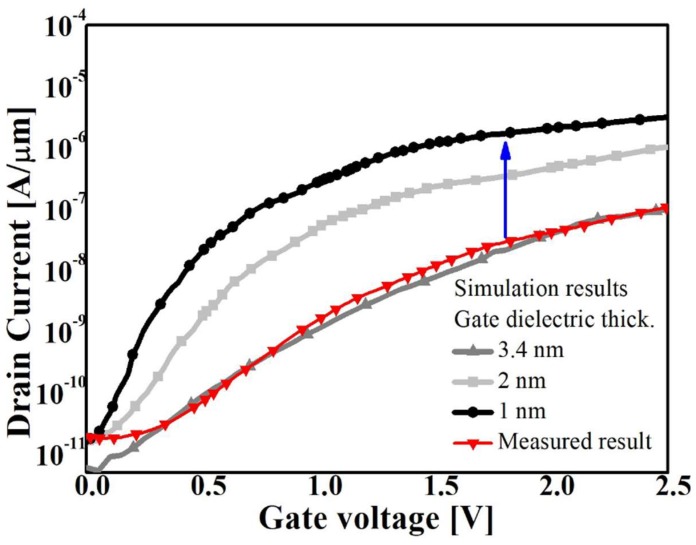
Transfer characteristics of the proposed TFETs with various CET.

**Figure 11:**
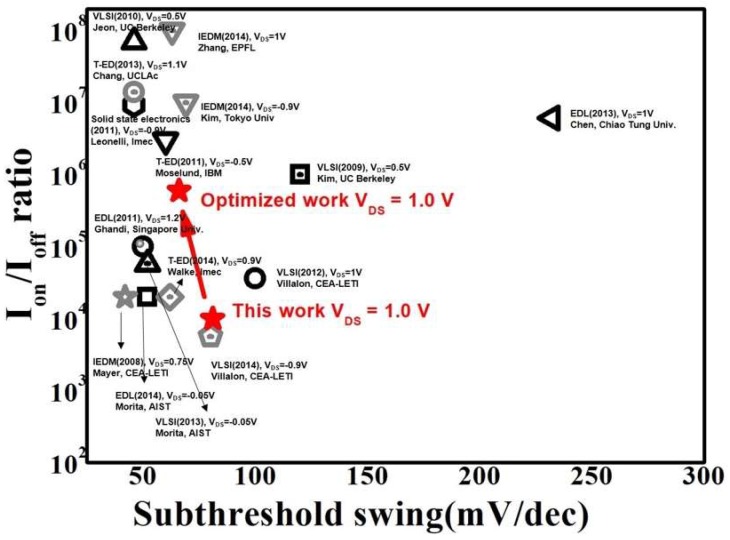
Performance comparison of TFETs. *I*_ON_/*I*_OFF_ of TFETs as a function.

The objective of this study is to demonstrate the TFET with high *I*_ON_ and low *I*_AMB_. Compared with planar TFETs which is fabricated with the same processes, there is no doubt that the proposed structure is effective to improve electrical performance. However, the measured results imply that it requires further optimization for the better performance than the other strategies [25,26,27,28,29,30,31,32,33,34,35,36,37]. Therefore, the proposed TFET’s feasibility for the better performance is examined by TCAD simulations using Synopsys Sentaurus™. Above all, BTBT parameters in Kane’s tunneling model are calibrated by measured results [17]. In the simulations, to calculate BTBT generation rate (*G*) per unit volume in the uniform electric field, Kane’s model is used and fitted parameters are as follows (Equation (1)).

(1) $$G=A{\left(\frac{F}{F_{0}}\right)}^{P}\exp\left(-\frac{B}{F}\right)\;$$

where *F*_0_ = 1 V/m, *P* = 2.5 for indirect BTBT. Prefactor *A* and exponential factor *B* are the Kane parameters and *F* is the electric field. Both linear and log scale simulated transfer characteristics are well matched to experimental data when *A*: 1 × 10^14^ cm^−1^·s^−1^/*B*: 3 × 10^6^ V/cm are applied to TFETs. Then, the thickness of the gate dielectric is analyzed. Unlike advanced technologies, the proposed TFET uses 3.4 nm thick SiO_2_ as the gate dielectric. Thus, if the gate dielectric is adjusted to 1 nm, the proposed TFET can obtain higher *I*_ON_ at the low *V*_GS_ (Figure 10). Figure 11 compares the *I*_ON_/*I*_OFF_ as a function of SS for the device shown in this paper and that in the previous articles [25,26,27,28,29,30,31,32,33,34,35,36,37]. Compared with the other Si based TFETs, the optimized TFET shows a remarkable performance in terms of minimum SS and *I*_ON_/*I*_OFF_.

## 5. Conclusions

In this paper, a novel TFET with SiGe fin channel and elevated drain has been introduced. The SiGe fin channel included small-bandgap and better electrostatic controllability which are leading high *I*_ON_ and low SS, compared to conventional planar TFETs. Furthermore, the elevated drain could yield lower *I*_AMB_ due to the increased physical distance between channel and drain. Considering these features, we have examined and demonstrated the fabrication processes of the proposed device. In addition, based on the measured results, the proposed TFET is calibrated by TCAD simulation. In order to optimize the device into state-of-the-art technique, the proposed device with thin gate dielectric is also simulated. The results proved that the device showed the improved *I*_ON_ current and smaller SS. Consequently, these features of the proposed device will be available for compensating the weaknesses of conventional TFETs. Therefore, it will be one of the promising candidates for next-generation devices.

## Figures and Tables

**Table 1:** Summary of extracted parameters.

	Si TFET	SiGe TFET	Propose TFET
SS (*V*_DS_ = 0.1 V)	151 mV/dec	87 mV/dec	81 mV/dec
*I*_ON_	4 nA/μm	21 nA/μm	139 nA/μm
*I*_AMB_	13 pA/μm	16 nA/μm	18 pA/μm
*I*_ON_/*I*_OFF_	4 × 10^3^	2.7 × 10^3^	2.8 × 10^4^
